# Development of the new CPTSD diagnosis for ICD-11

**DOI:** 10.1186/s40479-021-00148-8

**Published:** 2021-03-01

**Authors:** Andreas Maercker

**Affiliations:** Department of Psychology, Division Psychopathology and Clinical Intervention, University of Zurich, Binzmühlestr. 14/17, 8044 Zurich, Switzerland

**Keywords:** Complex post-traumatic stress disorder, ICD-11 development, Trauma- and stress-related disorders

## Abstract

**Background:**

The diagnosis of complex post-traumatic stress disorder (CPTSD) was proposed several decades ago by scientist-practitioners, almost parallel to the first description of the diagnosis of post-traumatic stress disorder (PTSD). In the previous International Classification of Diseases, version 10 (ICD-10) issued by the World Health Organization (WHO), this symptom constellation was termed ‘enduring personality change after catastrophic experience’. This diagnosis has not been clinically influential, nor has it been subjected to much research. Thus, in a multi-stage process of ICD-11 development, the diagnosis of CPTSD was developed.

**Methods:**

This paper provides a review of the historical lines of development that led to the CPTSD diagnosis, as well as the results since the ICD-11 publication in 2018.

**Results:**

The CPTSD diagnosis comprises the core symptoms of the – newly, narrowly defined – PTSD diagnosis, the three symptom groups of affective, relationship, and self-concept changes. The diagnosis is clinically easy to use in accordance with the WHO development goals for the ICD-11 and has shown good psychodiagnostic properties in various studies, including good discrimination from personality disorder with borderline pattern.

**Conclusion:**

The scholarly use of the new diagnosis has resulted in an increasing number of published studies on this topic in the diagnostic and therapeutic fields.

## History of PTSD

The introduction of the diagnosis of posttraumatic stress disorder (PTSD) in the Diagnostic and Statistical Manual, Version III (American Psychiatric Association, 1980) was a major milestone for the mental health field. An externally caused mental disorder was introduced into the state of the art of psychiatry and clinical psychology – a kind of scientific recognition, which has never been seen before in classification systems of mental disorders. The introduction of the diagnosis followed a political negotiation process in U.S. psychiatry, in which scientist-practitioners played an important role, with Vietnam veterans on the one hand and the women’s rights movement on the other hand as advocates [1, 2]. The Vietnam War had ended in 1975, and American Veterans Administration Hospitals were faced with large numbers of traumatized veterans they had to care for. The women’s rights movement could make its voice heard for traumatized women as victims of domestic or sexualized violence.

Just as important as the political advocacy was the further development of psychopathology or the investigation of psychological stress consequences at that time. Mardi J. Horowitz had presented the concept of ‘stress response syndromes’, which turned out to gain wide attention through clinically precise descriptions and a psychodynamic-cognitive model and was accompanied by a large empirical research program [3]. He described prototypically the psychological consequences of severe traffic accidents and applied this to wartime experiences, concentration camp imprisonment, rape, and life-threatening medical conditions. As core symptom groups, he depicted intrusions and avoidance, followed by negative cognitive and mood changes such as guilt and shame. This research-based and operationalized approach laid the scientific foundation for PTSD as a new disease entity [3].

## Prehistory of complex PTSD

In a milestone book, Judith Lewis Herman [4] summarized her clinical research with (female) victims of domestic and sexualized violence, including child sexual abuse. She proposed a new diagnosis, which she called complex post-traumatic stress disorder (CPTSD). This diagnostic proposal had six symptom groups: Disturbance of affect regulation, alterations of consciousness, disturbed self-perception, disturbed perception of the offender, relationship problems, and changes in the value system. At the same time, Herman [4] described a therapeutic framework approach that distinguishes three phases: security, remembering and grieving, and reconnection.

With a group of mainly child and adolescent psychiatrists and psychologists, van der Kolk [5] developed the concept of ‘Developmental Trauma Disorder’ (DTD) and proposed it for introduction into the to be developed DSM. For children and adolescents, it was proposed as a definition that “multiple or chronic exposure to one or more forms of developmentally adverse interpersonal trauma” ([5], p. 405) leads to a pattern of psychopathological changes. This is described as a “triggered pattern of repeated dysregulation in response to trauma cues [...], persistently altered attributions and expectancies [...], (and) functional impairment [...]” ([5], p. 405). The DTD concept has been empirically investigated in several international studies, which led to a mixed picture of the validity and usefulness of this approach [6].

The concept of ‘Disorders of Extreme Stress-Not otherwise specified’ (DESNOS) was developed in parallel for the appendix of DSM-IV (2003) with research diagnoses [7]. The operationalization largely followed Herman’s theoretically formulated CPTSD model [4]: Symptoms of affect dysregulation, dissociation, somatization, altered self-perception, altered relationships, and altered sustaining beliefs. It is still not entirely clear why research on DTD and the DESNOS concept did not lead to the inclusion of these diagnostic concepts, in the presented or modified form, in the DSM-5 in 2013. Resick et al. [8] concluded that there would still be too few empirical studies on these concepts that would provide the necessary validations of the concept. Thus, the internal coherence of the DESNOS concept had been considered insufficient by several studies ([e.g., [9]).

### Enduring personality change and further preparatory work

In 1990, the PTSD diagnosis was first officially recognized in the International Classification of Diseases, 10th version (ICD-10: Word Health Organization, 1990). In addition to PTSD, the chapter on ‘Disorders of adult personality and behaviour’ included the diagnosis ‘Enduring personality change after catastrophic experience’ (EPCACE: ICD-10 code F62.0). This disorder concept was based on the diagnostic proposal of a ‘concentration camp syndrome’ by Leo Eitinger [10]. However, this narrower model was abandoned in favor of a more general formulation. EPCACE was symptomatically defined in the ICD-10 research criteria by a persistent hostile or suspicious attitude towards the world, social withdrawal, a persistent feeling of emptiness or hopelessness, but not with the full core symptoms of PTSD.

EPCACE had, however, received minimal attention in the expert literature. One particular criticism concerned the lack of specificity of its criteria and the difficulty of using broadly defined sets of criteria in practice [11]. Not a single study or case report was devoted to this disorder in connection with childhood abuse or sexual violence.

To solve the basic psychometric validity problems of the assessments for complex presentations of trauma sequelae, Briere et al. [12] developed the Trauma Symptom Inventory as a self-report. The Trauma Symptom Inventory contained broad areas (ten symptom clusters and so-called validation scales) of possible trauma consequences and was examined in many samples of child abuse/maltreatment or sexual violence survivors. The results obtained with this instrument using elaborate methodology were used to formulate the ICD-11 definition of Complex PTSD. In particular, these data showed that patients with complex trauma episodes not only experienced affective, relationship, and self-image problems, but also showed the core symptoms of ‘classic’ PTSD, i.e. intrusions, avoidance, and hyperreactivity ([e.g., [13]).

A further milestone along the way to the current CPTSD formulation was the expert survey of the International Society for Traumatic Stress Studies on best practice treatment of Complex PTSD, in which 50 international experts were interviewed [14]. The results showed a preference for sequential treatment, a primary focus on coping skills (including emotion regulation interventions), and on the narration of trauma memory (using various therapeutic techniques). Thus, despite the existence of very few randomized therapy studies, a basic consensus on the most important therapeutic goals was documented.

### The ICD-11 process

The WHO had set the goal to increase the clinical utility of all diagnoses in the new ICD-11 (published in 2018), which was mainly to be achieved by the lowest possible number of core symptoms. This should enable clinicians in all parts of the world to use the diagnosis as easily as possible. In addition, new diagnoses should only be introduced if there is sufficient clinical knowledge for specific therapies. The working group for diagnoses in the area of ‘Specific Stress-related Disorders’, which was composed of members from all continents and various NGOs, decided that the PTSD diagnosis established since 1980 should be complemented by a sibling diagnosis, the complex PTSD diagnosis. This replaces the previous EPCACE diagnosis.

The core symptoms of classical PTSD have been narrowed down and are now: Re-experience in the present, avoidance of traumatic reminders, and a sense of current threat. These three symptom groups are also part of the CPTSD diagnosis. In CPTSD there are three additional symptom groups that can be summarized as disturbances in self-organization: Emotion regulation difficulties (e.g., problems calming down), relationship difficulties (e.g., avoidance of relationships) and negative self-concept (e.g., beliefs about the self as a failure) [15].

The work of the WHO work group included conducting several clinical field studies on the new concepts. First, validity aspects of the diagnoses were investigated in comparison to the previous diagnoses in an international case-controlled field study. It was found that the new CPTSD diagnosis with 83% inter-rater agreement was more correctly assessed by clinicians than EPCACE with 65% inter-rater agreement [16]. Subsequently, field studies in 13 countries with 340 clinicians and 1806 patients were conducted to verify the agreement of the evaluators. Here, the CPTSD diagnosis had a mean kappa = .56 [17] – which led to a further optimization of the narrative definition in the WHO Clinical Guidelines. As a result, in a subsequent web-based clinical study it was in the top group of several diagnoses for correct diagnosis (percentage of diagnostic accuracy) [18].

Of course, at all stages of the development of the CPTSD diagnosis in ICD-11, clinical differentiation from borderline personality disorder (BPD) played a role. In the meantime, some research exists that provides information on this distinction and point to the treatment implications of these differences, e.g. [19]. While the self-image of patients with BPD changes abruptly between exaggeratedly negative and exaggeratedly positive self-perceptions, in CPTSD it remains persistently negative. In BPD, the relationship difficulties show up with rapid relationship initiation and an up and down of idealization and devaluation of the partners, while CPTSD patients avoid or break off relationships in times of strong general stress. The two diagnoses also differ in terms of suicidal tendencies: In BPD, these suicidal tendencies occur together with self-harming behaviour and thus become a primary therapeutic goal, while in CPTSD the frequency and intensity of these problems is lower.

In the meantime, an international consortium of researchers and clinicians has developed a measurement tool—both a self-rating version and a clinician-assessment version—that assesses diagnosis and severity (www.traumameasuresglobal.com). Validated versions of the self-rating version are already available in different languages, while the validation of the clinician assessment in different languages is still in progress (see above website).

### Conclusion: strengthening CPTSD research

Since the publication of the beta version of the CPTSD definition by the ICD-11 working group [15], there has been a boom in research on this new diagnosis, especially in diagnostic and prevalence research. A PubMed® search in titles (search terms: [complex post-traumatic stress disorder or complex PTSD or CPTSD]) resulted in nine publications for 2014, which increased over all years (e.g., 2016: 16; 2018: 28; 2020: 31 so far). Reviews are available on the validity aspects of the CPTSD diagnosis – also in distinction to classic PTSD [20] and evidence-based treatment options [21]. It is obvious that research into the bio-psycho-social-cultural conditions of the disorder should be intensified, and this will certainly happen more intensively in the coming years.

## Data Availability

N.A.
